# Mixed‐phenotype acute leukemia consisting of five heterogeneous leukemic populations without the expression of CD34

**DOI:** 10.1002/jha2.86

**Published:** 2020-09-18

**Authors:** Takahiro Nishino, Yu Aruga, Chiaki Ikeda, Akiko Miyagi Maeshima, Dai Maruyama, Hiromichi Matsushita

**Affiliations:** ^1^ Department of Laboratory Medicine, National Cancer Centre Hospital Tokyo Japan; ^2^ Department of Pathology National Cancer Centre Hospital Tokyo Japan; ^3^ Department of Haematology National Cancer Centre Hospital Tokyo Japan

A 52‐year‐old man suffering from Crohn's disease presented with generalized lymphadenopathy and an anterior mediastinal mass with bilateral pleural effusion. Based on the lymph node biopsy findings, the patient was diagnosed with mixed‐phenotype acute leukemia (MPAL), T/myeloid, not otherwise specified, accompanied by the weak expression of CD19 and CD79a. A chromosomal analysis showed tetraploidy with a complex karyotype. He received acute lymphoblastic leukemia‐directed chemotherapy. However, the lymphadenopathy worsened and was accompanied by leukocytosis (14.8 × 10^9^/L) with the appearance of blastic cells (84%).

The blood film showed two blast populations (**Figure** 1 **Panels** **A and B**). One population was composed of myeloblast‐like cells that had fine chromatin formation with prominent nucleoli and a relatively abundant cytoplasm. Peroxidase staining of this population was positive. The medium‐sized cells possessed fine azurophilic granules in their cytoplasm, and granules in the large‐sized cells were more numerous and denser. The other population was composed of lymphoblast‐like cells that were small to medium in size with a rather coarse chromatin formation and scanty agranular cytoplasm.

Flow cytometry of the peripheral blood identified these two blast populations as side scatter (SSC)‐high (presented in green) and SSC‐low (presented in red) populations in CD45 weakly positive fraction, neither of which expressed CD34 (**Figure** 1 **Panel C, Figure S1**). The SSC‐high population, which commonly expressed myeloid markers, including CD13, CD33, and myeloperoxidase (MPO), as well as terminal deoxynucleotidyl transferase (TdT), were further divided into two populations: cyCD3‐positive (T‐lineage/myeloid) and cyCD3‐negative (myeloid). The SSC‐low population commonly expressed T‐lymphoid markers as well as myeloid markers was also further divided into three populations: T‐lineage expressing B‐cell markers (cyCD3+/cyCD79a w+ or sCD3+/CD19w+), T‐lineage (cyCD3+/cyCD79a− or sCD3+/CD19−), and a B‐cell marker expressing population (cyCD3−/cyCD79a w+ or sCD3−/CD19 w+).

It is hypothesized that the normal counterparts of MPAL are multipotent hematopoietic stem cells. Five leukemic bipotent or lineage‐committed populations detected in this rare MPAL case might reflect the hierarchical development and plasticity of leukemic cells with multipotent capacity, although they showed developmental maturity, as represented by the loss of the CD34 expression.

**FIGURE 1:**
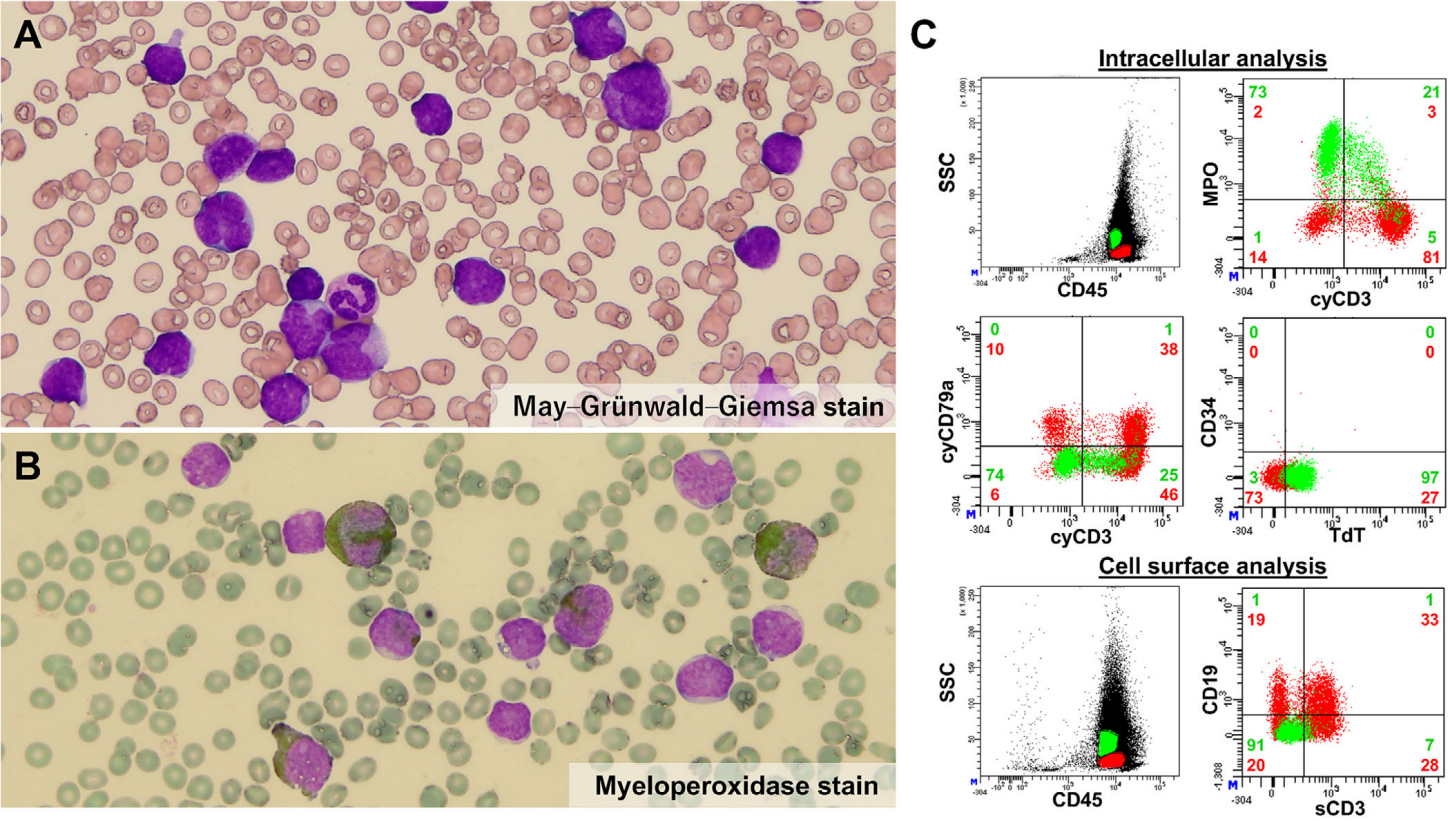
MPAL consisting of 5 heterogeneous leukaemic populations without the expression of CD34. A and B, The blood film with May‒Grünwald‒Giemsa stain (A) and Myeloperoxidase stain (B), C, Flow cytometry of the peripheral blood.

## CONFLICT OF INTEREST

The authors declare that there is no conflict of interest.

## AUTHOR CONTRIBUTIONS

Takahiro Nishino performed the laboratory analyses and wrote the manuscript. Yu Aruga and Chiaki Ikeda performed the laboratory analyses. Akiko Miyagi Maeshima performed the histopathological and immunohistochemical analyses and reviewed the manuscript critically. Dai Maruyama diagnosed and managed the case and reviewed the manuscript critically. Hiromichi Matsushita wrote the manuscript and approved the submission of the final version.

## Supporting information

Figure S1

