# Supplementary figures and images for: Mixed‐phenotype acute leukemia consisting of five heterogeneous leukemic populations without the expression of CD34
Source: EJHaem. 2020 Sep 18;1(2):406–7. doi: 10.1002/jha2.86 (PMC9176053; doi:10.1002/jha2.86)

figure S1

## Cell surface analysis

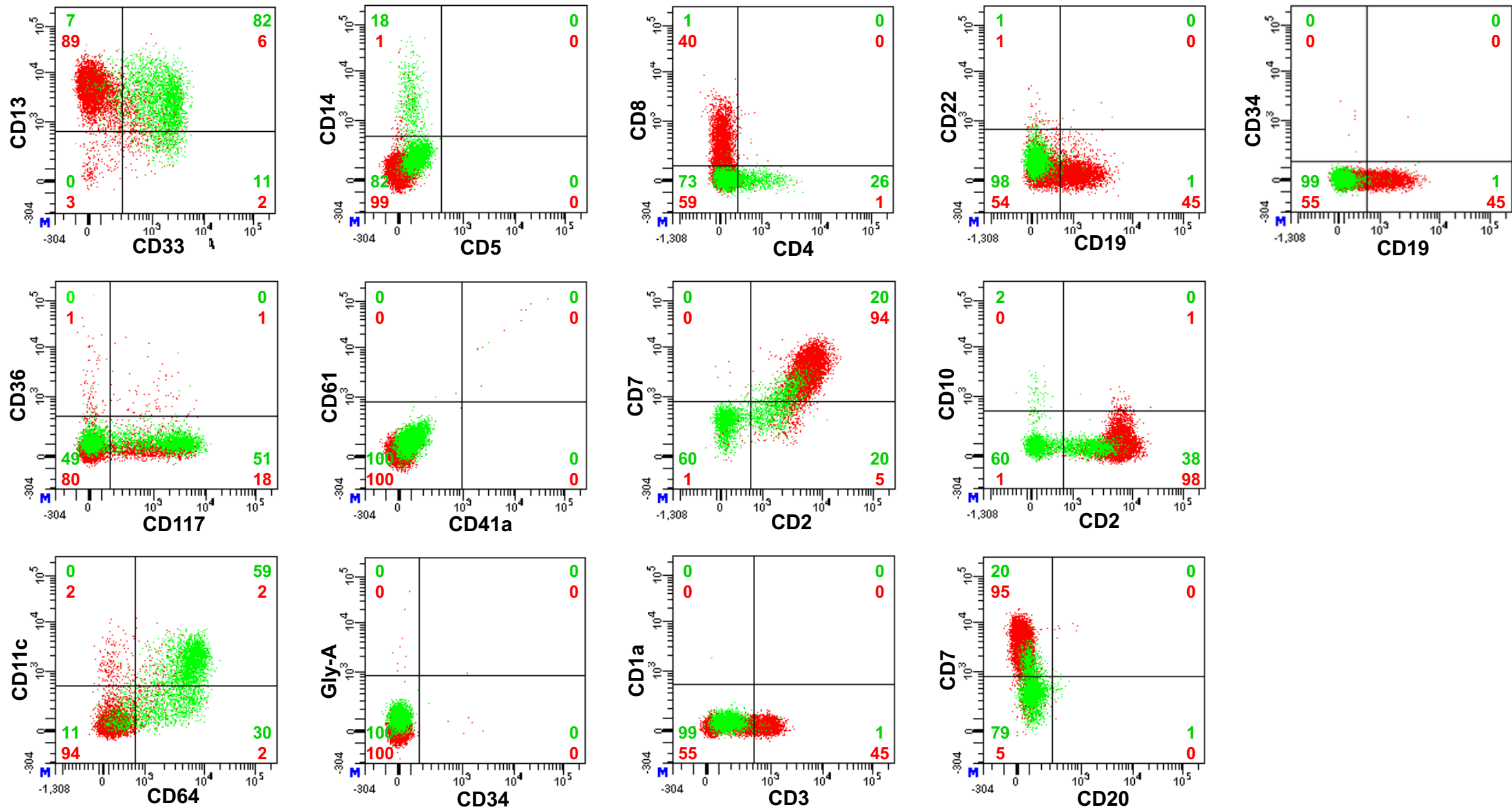

Supplement: Supplementary file 1 — Figure S1 [file JHA2-1-406-s001.pdf]
